# A Single ssRNA Segment Encoding RdRp Is Sufficient for Replication, Infection, and Transmission of Ourmia-Like Virus in Fungi

**DOI:** 10.3389/fmicb.2020.00379

**Published:** 2020-03-18

**Authors:** Qihua Wang, Fan Mu, Jiatao Xie, Jiasen Cheng, Yanping Fu, Daohong Jiang

**Affiliations:** ^1^State Key Laboratory of Agricultural Microbiology, Huazhong Agricultural University, Wuhan, China; ^2^Key Laboratory of Plant Pathology, College of Plant Science and Technology, Huazhong Agricultural University, Wuhan, China

**Keywords:** *Sclerotinia sclerotiorum*, *Botourmiaviridae*, ourmia-like virus, mycovirus, transfection

## Abstract

Recently, an increasing number of ourmia-like viruses have been found in fungi; however, the features of these viruses remain unknown. Here, we report a novel ourmia-like virus isolated from *Sclerotinia sclerotiorum*. This virus, named *S. sclerotiorum* ourmia-like virus 4 (SsOLV4), has a genome 2,982 nt in length with a G-pentamer (GGGGG) at the 5′-terminus and a C-pentamer (CCCCC) at the 3′-terminus. The SsOLV4 genome has only one large putative open reading frame (ORF) predicted with both standard codes and mitochondrial codes and encodes an RNA-dependent RNA polymerase (RdRp). SsOLV4 is closely phylogenetically related to *Pyricularia oryzae* ourmia-like virus 1, with 42% identity between the RdRp amino acid sequences. We constructed full-length cDNA of SsOLV4 and synthesized RNA *in vitro* using the T7 RNA polymerase. The synthesized RNA could transfect *S. sclerotiorum* protoplasts efficiently. We further found that viral RNA could infect mycelia when mixed with PEG buffer. Our study suggests that a novel genus in family *Botourmiaviridae* should be established for SsOLV4 and other related viruses and demonstrates that one single-stranded RNA segment encoding RdRp is sufficient for ourmia-like viruses in fungi.

## Introduction

Mycoviruses, which reside in fungi, are widespread in all major fungal taxa in nature (Ghabrial et al., 2015) and can be grouped into 19 families as described by the International Committee on Taxonomy of Viruses (ICTV). Currently, an increasing number of mycoviruses have been identified through metatranscriptomics (Marzano et al., 2016). Continuous discoveries of novel mycoviruses have expanded our knowledge of viruses (Chiba and Suzuki, 2015; Kanhayuwa et al., 2015; Zhang et al., 2016). Mycoviruses are often phylogenetically related to viruses that infect other organisms; thus, the identification of novel mycoviruses may also help us to understand the evolution and ecology of viruses. Some mycoviruses are most closely phylogenetically related to plant viruses and have been suggested to originally derive from plant viruses. Fungi may acquire viruses when living on virus-infected plants (Howitt et al., 2001, 2006; Xie et al., 2006). Mascia et al. (2014) reported that tobacco mosaic virus (TMV) could replicate and even persist in the *Colletotrichum acutatum*. More recently, Andika et al. (2017) found that cucumber mosaic virus (CMV) could be transferred between plants and fungi. Nerva et al. (2017) presented strong evidence to show that a virus that infects an endophytic fungus could replicate in plants. Additionally, viroids have been shown to infect fungi efficiently (Wei et al., 2019). These findings further confirmed that some mycoviruses are close relatives to plant viruses.

Viruses in family *Narnaviridae* have a naked single-stranded RNA (ssRNA) genome and are grouped into two genera, *Narnavirus* and *Mitovirus*. *Narnaviridae* was originally identified in fungi (Rodriguez-Cousiño et al., 1991; Polashock and Hillman, 1994). It is believed that genes of mitovirus have been integrated into the genome of plants (Bruenn et al., 2015), suggesting that mitoviruses may have once invaded plants (Nibert et al., 2018). *Chenopodium quinoa* mitovirus 1 was confirmed to infect mitochondria of plants (Nerva et al., 2019). Recently, viruses phylogenetically related to *Narnaviridae* or *Ourmiavirus* were identified from insects or protozoa via next-generation sequencing (NGS) (Shi et al., 2016; Sukla et al., 2017), suggesting that narnavirus or narna-like viruses are widespread in other organisms. Viruses in the *Narnaviridae* family have a distant phylogenetic relationship with *Ourmiavirus* based on the RNA replicase sequence (Rastgou et al., 2009). The first ourmiavirus was isolated from a diseased melon and named ourmia melon virus (OuMV) (Lisa et al., 1988); then two ourmiaviruses (cassava virus C and Epirus cherry virus) were discovered in other plants (Avgelis et al., 1989; Fauquet et al., 2005); thus, the virus genus *Ourmiavirus* was established. Members of the genus *Ourmiavirus* typically contain three genomic RNA segments, with a unique bacilliform virion structure (Brown, 1997; Hull, 2014). Recently, several mycoviruses that are phylogenetically related to ourmiaviruses have been identified (Donaire et al., 2016; Hrabakova et al., 2017; Illana et al., 2017; Li et al., 2019; Ohkita et al., 2019). Therefore, ICTV has created a new family (*Botourmiaviridae*) to adapt these ourmia-like viruses (classified into three genera) and existing ourmiaviruses. Interestingly, only one large RNA segment encoding an RNA replicase has been identified in all of these fungal ourmia-like viruses; whether these fungal ourmia-like viruses have other genomic RNA segments remains unknown.

Although mycoviruses were identified in fungi 54 years ago, transfection methods and reverse genetic systems have been developed only for a few viruses (Son et al., 2015). Previously, infectious cDNA clones were constructed in several mycoviruses, namely, *Cryphonectria hypovirus 1* (Choi and Nuss, 1992), *Diaporthe RNA virus 1* (Moleleki et al., 2003), *Saccharomyces 23S RNA narnavirus* (Esteban and Fujimura, 2003), *Saccharomyces 20S RNA narnavirus* (Esteban et al., 2005), *Sclerotinia sclerotiorum hypovirus 2* (Marzano et al., 2015), and yado-nushi virus 1 and yado-kari virus 1 (Zhang et al., 2016).

*Sclerotinia sclerotiorum* (Lib.) is a devastating pathogen that can infect many economically important crops and cause fatal white mold and soft rot (Bolton et al., 2006). In this study, an ssRNA mycovirus (SsOLV4) encoding an RNA-dependent RNA polymerase (RdRp) similar to the ourmia-like viruses was identified from *S. sclerotiorum* through a novel metatranscriptomic approach. The SsOLV4 sequence was analyzed, and the viral infectious ability was measured.

## Results

### Viruses in *S. sclerotiorum* Strain PX14A4 and Its Relatives

Two *S. sclerotiorum* strains, named PX14A1 and PX14A4, were isolated from a sclerotium collected in Sichuan Province, China. Total RNA from each strain was extracted and mixed equally to perform NGS. The assembled sequences based on NGS reads were used for homology searches against the National Center for Biotechnology Information (NCBI) virus amino acid sequence database using BLASTX. From this database, we identified 47 contigs belonging to nine viruses, including six contigs that could be assembled into one long contig, which was similar to the sequence of *Phomopsis longicolla* RNA virus 1 (PlRV1, GenBank accession number YP_009345044.1). We temporarily named this virus *Sclerotinia sclerotiorum ourmia-like virus 4* (SsOLV4, GenBank accession no. MN715322).

**FIGURE 1:**
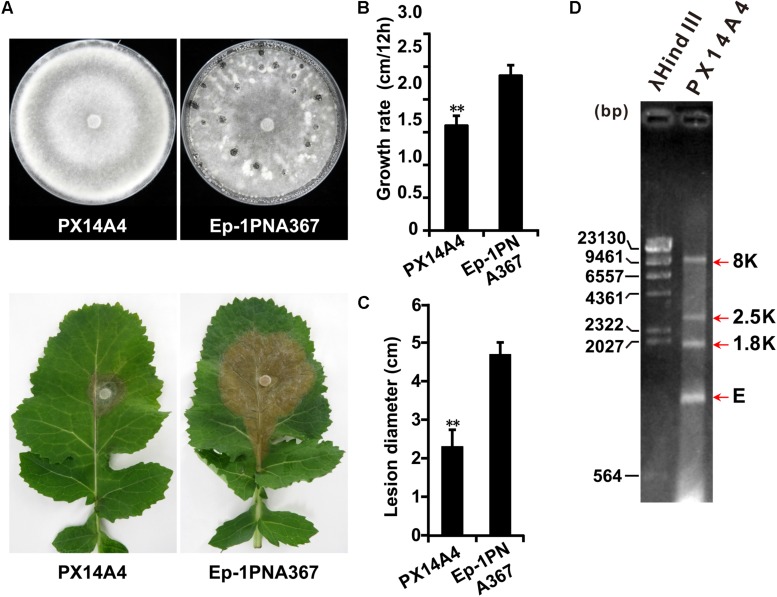
The biological characteristics and dsRNA pattern of *Sclerotinia sclerotiorum* strain PX14A4. **(A)** Colony morphology of strain PX14A4 and virulent strain Ep-1PNA367 (on PDA for 14 days at 21°C) (top). Lesion induced by strain PX14A4 or Ep-1PNA367 on detached rapeseed leaves (72 h post inoculation under 21°C) (bottom). Growth rates **(B)** and average lesion diameters **(C)** of strains PX14A4 and Ep-1PNA367. “**” indicates a significant difference (*p* < 0.01) between strain PX14A4 and Ep-1PNA367. **(D)** Agarose gel electrophoresis analysis of the dsRNA extracted from strain PX14A4. The dsRNA was treated with DNase I and S1 nuclease prior to electrophoresis. The dsRNA was named 8K, 2.5K, 1.8K, and E. Size of the DNA ladder standard is indicated in base pair (bp). M, molecular weight marker (Takara).

Both strains PX14A1 and PX14A4 harbor SsOLV4, and strain PX14A4 was chosen for further study. Strain PX14A4 has a slower hyphal growth rate and cannot produce sclerotia on potato dextrose agar (PDA) medium, and it could only induce lesions that were much smaller than those of the virulent strain Ep-1PNA367 (virus free) on the detached rapeseed leaves (Figures 1A–C). The double-stranded RNA (dsRNA) was extracted from strain PX14A4 and treated with DNase I and S1 nuclease to digest the genomic DNA and ssRNA. We found that strain PX14A4 harbors four dsRNA segments that were named 8K, 2.5K, 1.8K, and E (Figure 1D).

### Genome Characteristics of SsOLV4

**FIGURE 2:**
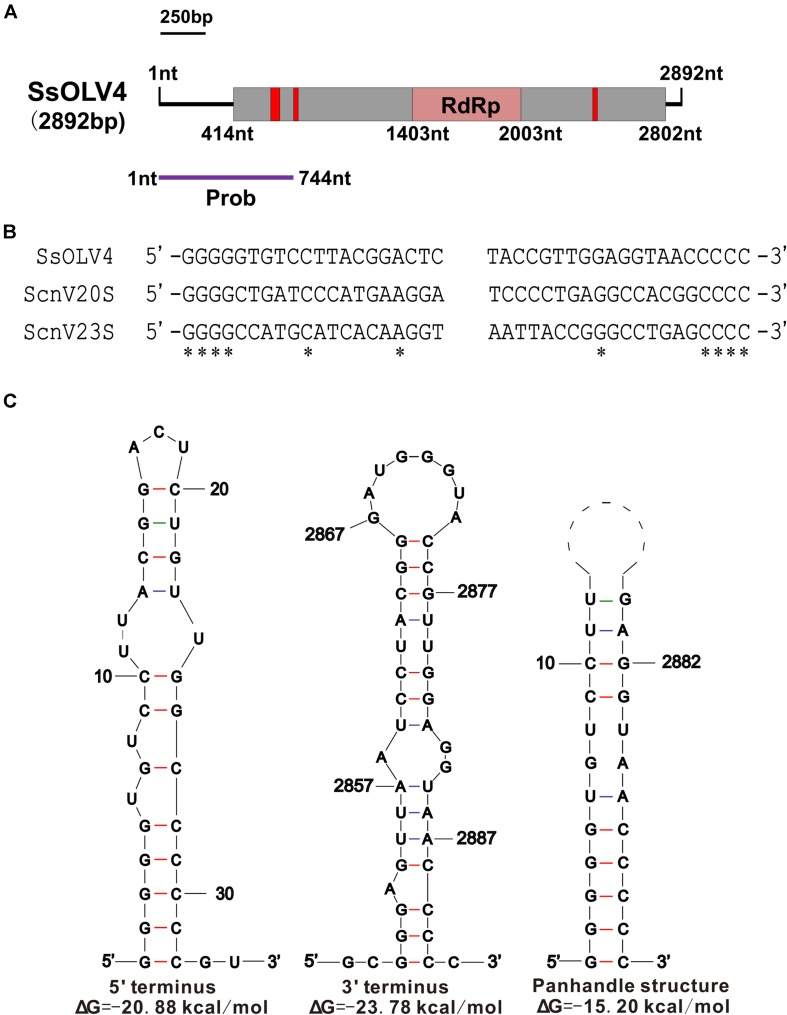
The genome organization and the terminal structure of SsOLV4. **(A)** A schematic diagram of the genome organization of SsOLV4. SsOLV4 contains a single open reading frame (ORF) shown as a gray box (414–2,802 nt), which putatively encodes the RNA-dependent-RNA polymerase (RdRp) domain, shown as a pink box (1,403–2,003 nt). The predicted nuclear location signal peptide sequences are shown as red boxes. The DNA segment amplified by RT-PCR was used as a probe in northern hybridization experiments and is shown as a purple line. Nt, nucleotide position on the genome. **(B)** Alignment of the 5′- and 3′-end regions of SsOLV4 and the two yeast narnaviruses ScnV20S (*Saccharomyces* 20S RNA narnavirus) and ScnV23S (*Saccharomyces* 23S RNA narnavirus); asterisks indicate the same nucleic acid. **(C)** Potential secondary structures of the 5′- (left) and 3′-termini (middle) of SsOLV4 and a putative panhandle structure (right) formed by the inverted complementarity at two terminal sequences are depicted; the ΔΔG values (kcal/mol) were calculated using Mfold RNA structure software.

We obtained the 5′- and 3′-terminal sequences of SsOLV4 using the classic RACE technique, and the full-length cDNA sequence of SsOLV4 was confirmed by RT-PCR. The sequence of SsOLV4 is 2,892 bp in length with a 46.5% G+C content. This length is similar to that of the members in family *Narnaviridae* and that of the largest segments of ourmiaviruses. The genome of SsOLV4 contains only one large ORF from positions 414 to 2802 based on the universal genetic code or mold genetic code, encoding a protein with 796 aa (89 kDa) (Figure 2A). The termini of SsOLV4 harbor a G-pentamer (GGGGG) at the 5′-terminus and a C-pentamer (CCCCC) at the 3′-terminus, which is similar to the narnaviruses in *Saccharomyces* (Figure 2B). The putative 5′-untranslated region (UTR) of SsOLV4, as long as 413 nt, is similar to the 5′-UTR (624 nt) of PlRV1, but much longer than that of viruses in genera *Ourmiavirus* and *Narnavirus* (Table 1). The 5′- and 3′- termini of SsOLV4 were predicted using Mfold RNA structure software, and the results showed they could form complex secondary structures. The proximal 1–34 nt of 5′-UTR and 3′ 2,847–2,892 nt can form stable RNA loops (Figure 2C, left and middle). A potential panhandle structure was predicted with a ΔG value of -15.20 kcal/mol based on the two terminus sequences (Figure 2C, right). The stem–loop and panhandle are typical structures in *Narnaviridae* (including mitovirus) and ourmiavirus.

**TABLE 1:** The terminal sequences of some ourmia-like viruses, ourmiaviruses, and narnaviruses.

**Clade**	**Virus name**	**5′-UTR length**	**3′-UTR length**	**Terminal sequence (5′ to 3′)**	**Accession no.**	**Ref. of SRA**
*Botourmiaviridae*	SsOLV4	413	90	GGGGGTGTCC—GGTAACCCCC		This research
*Botourmiaviridae*	*Aspergillus neoniger* ourmia-like virus 1	335	63	CCGTTTGTTA—AAAGGCCTGC	MK279481.1	Gilbert et al., 2019
*Botourmiaviridae*	*Pyricularia oryzae* ourmia-like virus 1	452	81	GGGGTCAGGG—GGGGTCAGGG	LC413501.1	Ohkita et al., 2019
*Botourmiaviridae*	*Phomopsis longicolla* RNA virus 1	624	71	TGCACGGGTT—CTGCCTGTTA	NC_033729.1	Hrabakova et al., 2017
*Botourmiaviridae*	*Botrytis* ourmia-like virus	41	693	CCCCATGTACC—CGCTCGGGGG	NC_028476.1	Donaire et al., 2016
*Ourmiavirus*	Ourmia melon virus	14	217	CCCGATTATC—GAAATCTGGG	NC_011068.1	Rastgou et al., 2009
*Ourmiavirus*	Cassava virus C	20	107	CCCAATTTTG—GAGAATCGGG	NC_013111.1	Rastgou et al., 2009
*Ourmiavirus*	Epirus cherry virus	29	181	CCCAGAATTC—GAAAACTGGG	NC_011065.1	Rastgou et al., 2009
*Narnavirus*	*Saccharomyces* 20S RNA narnavirus	12	12	GGGGCTGATC—CCACGGCCCC	NC_004051.1	Rodriguez-Cousino et al., 1998
*Narnavirus*	*Saccharomyces* 23S RNA narnavirus	6	59	GGGGCCATGC—CCTGAGCCCC	NC_004050.1	Rodriguez-Cousino et al., 1998
*Mitovirus*	*Nigrospora oryzae* mitovirus 1	353	141	GGGTTTACCG—GAGAATACCC	MH823901.1	Liu, Submission
*Mitovirus*	*Fusarium boothii* mitovirus 1	194	138	GGGGAAACGA—AGAATGCCCC	LC425114.1	Mizutani et al., 2018
*Mitovirus*	*Sclerotinia nivalis* mitovirus 2	291	155	GGGGACCAAC—CTTATACCCC	KT365896.1	Wu, unpublished

**FIGURE 3:**
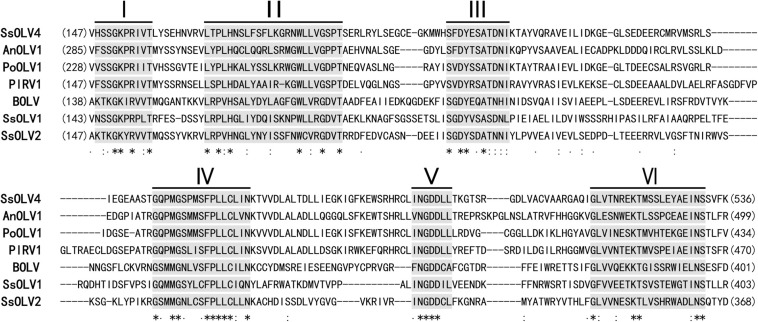
Multiple alignment of conserved amino acid motifs in RdRp regions of SsOLV4 and other selected ourmia-like viruses. The alignment was performed using ClustalX (2.0). The conserved motifs in RdRp of the compared viruses are shown by Roman numerals I to VI. “^∗^” indicates a same amino acid (aa) position; “:” indicates that one of the following ŚstrongŠ groups is fully conserved; “.” indicates that one of the following ‘weaker’ groups is fully conserved. Abbreviations of virus are listed in Table 2.

The ORF-encoded protein contained one conserved domain (RdRp). When the full-length SsOLV4 RdRp was analyzed using Position-Specific Iterated BLAST, up to 52% identity to other ourmia-like mycoviruses was detected (Table 2). Furthermore, a search of the Conserved Domain Database (CDD) and multiple protein sequence alignments suggested that the predicted RdRp domain contains six conserved motifs (Figure 3). Interestingly, we found three predicted monopartite nuclear localization signals (NLSs) and one bipartite NLS profile in viral predicted protein using the online software cNLS Mapper (Supplementary Table S2)^1^.

**TABLE 2:** Identity between the RdRp of SsOLV4 and those of ourmia-like mycoviruses.

**Virus name**	**Abbreviation**	**Accession no.**	**Query cover**	**Identities**	**Positives**	***E*-value**
*Penicillium sumatrense* ourmia-like virus 1	PsOulV1	QDB75000.1	62%	316/609 (52%)	393/609 (64%)	0
*Epicoccum nigrum* ourmia-like virus 2	EnOulV2	QDB75005.1	57%	295/552 (53%)	379/552 (68%)	0
*Cladosporium uredinicola* ourmiavirus 2	CuOulV2	QDB75002.1	57%	295/552 (53%)	377/552 (68%)	0
*Aspergillus neoniger* ourmia-like virus 1	AnOLV1	AZT88620.1	68%	245/582 (42%)	326/582 (56%)	3e−102
*Pyricularia oryzae* ourmia-like virus 1	PoOLV1	BBF90576.1	70%	236/562 (42%)	320/562 (56%)	7e−100
*Phomopsis longicolla* RNA virus 1	PlRV1	YP_009345044.1	78%	243/644 (38%)	354/644 (54%)	5e−112
*Sclerotinia sclerotiorum* ourmia-like virus 3	SsOLV3	AWY11006.1	41%	100/360 (28%)	162/360 (45%)	1e−17
*Sclerotinia sclerotiorum* ourmia-like virus 2 RNA 1	SsOLV2	ALD89139.1	38%	98/337 (29%)	151/337 (44%)	2e−16
*Botrytis ourmia*-like virus	BOLV	YP_009182165.1	41%	96/353 (27%)	157/353 (44%)	2e−16
*Sclerotinia sclerotiorum* ourmia-like virus 1 RNA 1	SsOLV1	ALD89138.1	46%	95/405 (23%)	168/405 (41%)	3e−12
*Aspergillus fumigatus* mitovirus 1	AfMV1	AXE72932.1	28%	72/244 (30%)	113/244 (46%)	4e−12
*Penicillium citrinum* ourmia-like virus 1	PcOLV1	AYP71797.1	28%	68/234 (29%)	107/234 (45%)	2e−11
Soybean leaf-associated ourmiavirus 1	SlaOV1	ALM62238.1	26%	68/226 (30%)	99/226 (43%)	6e−10
*Rhizoctonia solani* ourmia-like virus 1 RNA 1	RsOLV1	ALD89131.1	28%	69/237 (29%)	106/237 (44%)	1e−09
*Magnaporthe oryzae* ourmia-like virus	MoOLV	SBQ28480.1	28%	62/231 (27%)	103/231 (44%)	2e−08
Soybean leaf-associated ourmiavirus 2	SlaOV2	ALM62250.1	32%	74/286 (26%)	118/286 (41%)	5e−08

### Phylogenetic Characteristics of SsOLV4

**FIGURE 4:**
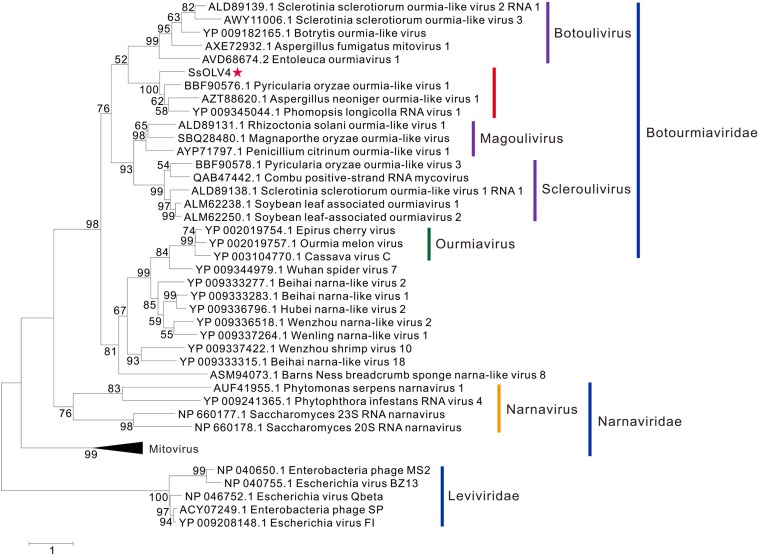
Maximum likelihood phylogenetic tree based on the RdRps of SsOLV4 and other related viruses. Proteins were aligned with MUSCLE implemented in MEGA 7. The multiple alignments of RdRp regions were based on the RdRp region of SsOLV4. The phylogenetic tree was generated using the software MEGA7. The bootstrap values (%) obtained with 1,000 replicates are indicated on the branches. Branches with bootstrap values of <50 have been collapsed. The scale bar at the lower left corresponds to a genetic distance of 1. SsOLV4 is marked by a red star. The lines with different colors represent the clade of each taxonomic status. The accession number in parentheses is in front of the virus name.

The phylogenetic analysis was performed using MEGA7 by the maximum likelihood method based on a Poisson model with 1000 bootstrap iterations for complete protein sequences of RdRp from narnaviruses, mitoviruses, ourmiaviruses, and phages in *Leviviridae*. SsOLV4 was grouped to the newly established family *Botourmiaviridae*. To date, the virus taxonomy of ICTV (2018b.v2) shows that *Botourmiaviridae* includes four genera, *Botoulivirus*, *Magoulivirus*, *Scleroulivirus*, and *Ourmiavirus* (Walker et al., 2019). SsOLV4 is closely related to genus *Botoulivirus*; however, SsOLV4 clustered with *Pyricularia oryzae* ourmia-like virus 1, *Aspergillus neoniger* ourmia-like virus 1, and *P. longicolla* RNA virus 1 and formed a phylogenetic branch independent of genus *Botoulivirus* (Figure 4).

### Transfection of *S. sclerotiorum* With *in vitro* Synthesized Viral RNA

**FIGURE 5:**
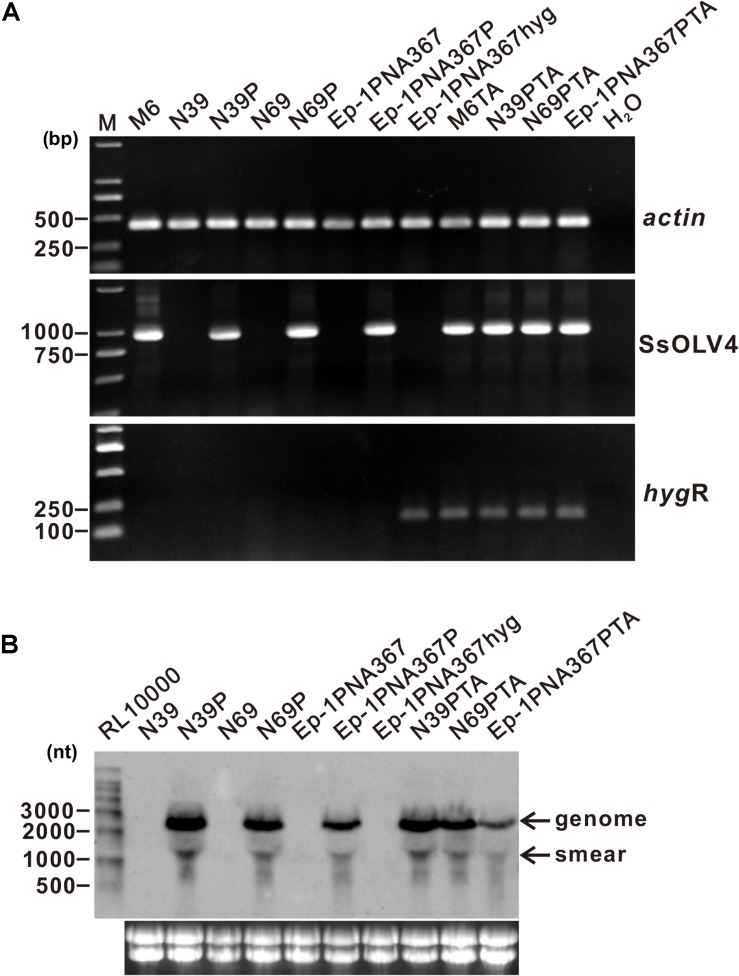
Detection of the synthesized viral RNA in the strains transfected using protoplasts as the receptor. **(A)** SsOLV4 RNA was detected by RT-PCR amplification from the transfected subcultures. Total RNA of each strain was used to synthesize cDNA. Gene *actin* was amplified as an internal control using primers ActinF and ActinR. Primers are listed in Supplementary Table S1. DNA was stained with ethidium bromide. Size of the DNA ladder standard is indicated in base pairs. **(B)** Northern hybridization to confirm the presence of SsOLV4. Total RNA from the strains was extracted from *S. sclerotiorum* mycelia grown for 2 days on PDA covered with cellophane. Each RNA sample was about 20 ng in total. Marker RL10000 (Takara) was detected using probe control DNA in alkaline Direct Labeling System (GE Healthcare). Lower panels are ethidium bromide-stained gels showing rRNA. The probe (location shown in Figure 2A) was synthesized with primers OMVFA and OMVR2 using PCR and directly labeled with alkaline phosphatase and detected by CDP-Star (GE).

To confirm the causal relationship between the virus and the host phenotype, we tried to eliminate viruses from strain PX14A4 by protoplast regeneration. Confirmed by RT-PCR, regenerated strain M6 contained only SsOLV4, while N39 and N69 lost all viruses identified (Figure 5).

We constructed a pMD18-P1 plasmid that contained the full-length cDNA of SsOLV4 (Supplementary Figure S1) and used the *Bam*HI linearized vector to produce artificial viral RNA “BAM” with T7 RNA polymerase (Supplementary Figure S2B). The artificial viral RNA “BAM” was used to transfect strains N39, N69, and Ep-1PNA367 using a PEG-mediated technique (Supplementary Figure S3B), and strains N39P, N69P, and Ep-1PNA367P were obtained.

**FIGURE 6:**
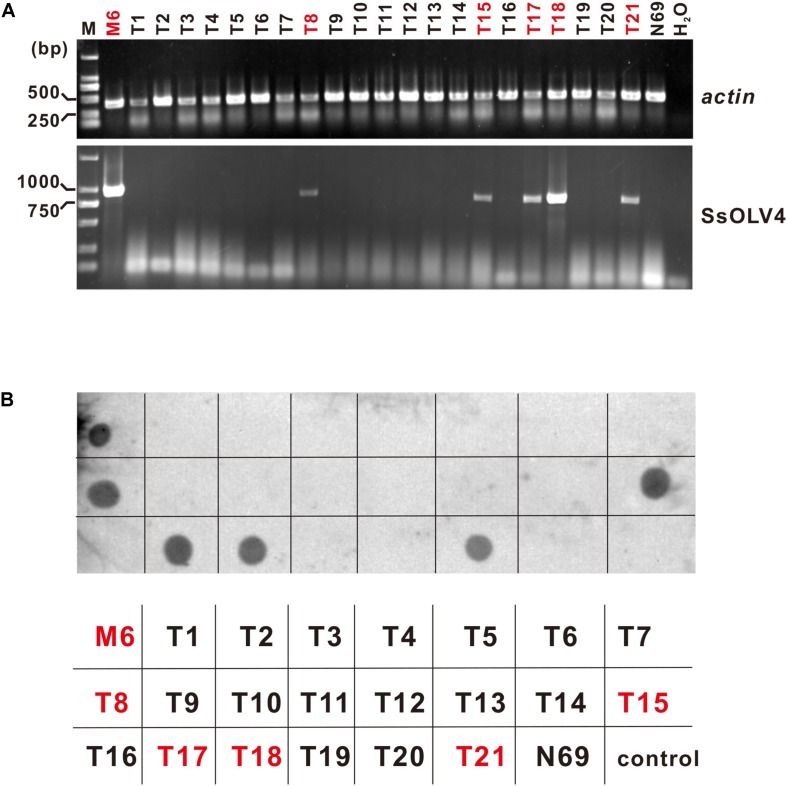
Detection of synthesized SsOLV4 RNA in the strains transfected using mycelia as the receptor. As described in the section “Materials and Methods,” the linearized vector pMD18-P1 was used to synthesize SsOLV4 RNA. The synthesized viral RNA was mixed with PEG buffer and added onto tips of fresh mycelia of strain N69. The infection experiments were performed on 21 colonies of strain N69. Each colony treated (named as T1 to T21) was subcultured three times (for about 10 days). The total RNA of all strains obtained was isolated to detect SsOLV4 using RT-PCR **(A)** and dot blot **(B)**. The RT-PCR primers and dot hybridization probe were the same as used in Figure 5. Total RNAs of strains M6 and N69 were used as positive and negative controls, and water (H_2_O) was used as a blank control. In **(B)**, the parallelism strain name was listed under the dot hybridization result. Strains infected with SsOLV4 were indicated in red.

SsOLV4 in strains N39P, N69P, and Ep-1PNA367P was confirmed by RT-PCR and northern hybridization. The results showed that viral RNA could be detected in strains N39P, N69P, and Ep-1PNA367P (Figure 5). The protoplast transfection efficiency was 100%. We also tried to transfect fungal mycelia with synthesized RNA (Supplementary Figure S3A), and 5 out of 21 subcultures were confirmed to adapt SsOLV4 (Figure 6). The results clarify that a single ssRNA segment encoding RdRp is sufficient for the ourmia-like virus to replicate in *S. sclerotiorum*.

### Synthesized Virus in the Transfected Strains

The *Bam*HI linearized vector pMD18-P1 template contained excess sequence in the 3′-terminus. In order to study the effect of the excess sequence, two synthesized viral RNAs with different lengths of 3′-terminus were constructed. The synthesized viral RNA with a shorter excess sequence was constructed using *Spe*I linearized vector pMD18-P1 as a template, and a transfected strain Ep-1PNA367SPE was obtained. The synthesized viral RNA with no excess sequence was constructed using the PCR product of pMD18-P1 amplified with primers M13 (+)/OMVRA as a template, and the transfected strain Ep-1PNA367RA was obtained. All strains Ep-1PNA367P, Ep-1PNA367SPE, and Ep-1PNA367RA were proven to harbor SsOLV4 (Supplementary Figure S4A).

The viral RNAs in strains Ep-1PNA367P, Ep-1PNA367SPE, and Ep-1PNA367RA were sequenced, and only two (in strains Ep-1PNA367SPE and Ep-1PNA367RA) or three (in strain Ep-1PNAP) nucleotides were alternated compared to the original template cDNA in pMD18-P1 (Supplementary Figure S4B), causing no frameshift mutation or translation termination. The termini were the same as the wild-type virus (SsOLV4 in strain M6). The results suggest that the synthesized viral RNAs remained stable.

### Synthesized Virus Could Be Transmitted Through Hyphal Anastomosis

In order to check whether the synthesized SsOLV4 could transmit among *S. sclerotiorum*, strains M6, N39P, N69P, and Ep-1PNA367P were dual cultured with the SsOLV4-free strain Ep-1PNA367hyg (labeled with a hygromycin B-resistant gene) side by side on the same PDA plate for 7 days. Then, the hyphal agar disks were taken from the Ep-1PNA367hyg colony and placed on hygromycin-amending PDA plates. The newly developed hygromycin-resistant subcultures (named M6TA, N39PTA, N69PTA, and Ep-1PNA367PTA) were subjected to SsOLV4 detection by RT-PCR amplification. The result showed that SsOLV4 could be successfully detected in all the newly developed colonies (Figure 5). Therefore, the wild-type SsOLV4 (in regenerant M6) and rescued SsOLV4 (in strains N39P, N69P, and Ep-1PNA367P) can transfer through hyphal anastomosis, and the replication of rescued SsOLV4 is stable.

### SsOLV4 Exists in Both Cytoplasm and Mitochondria

**FIGURE 7:**
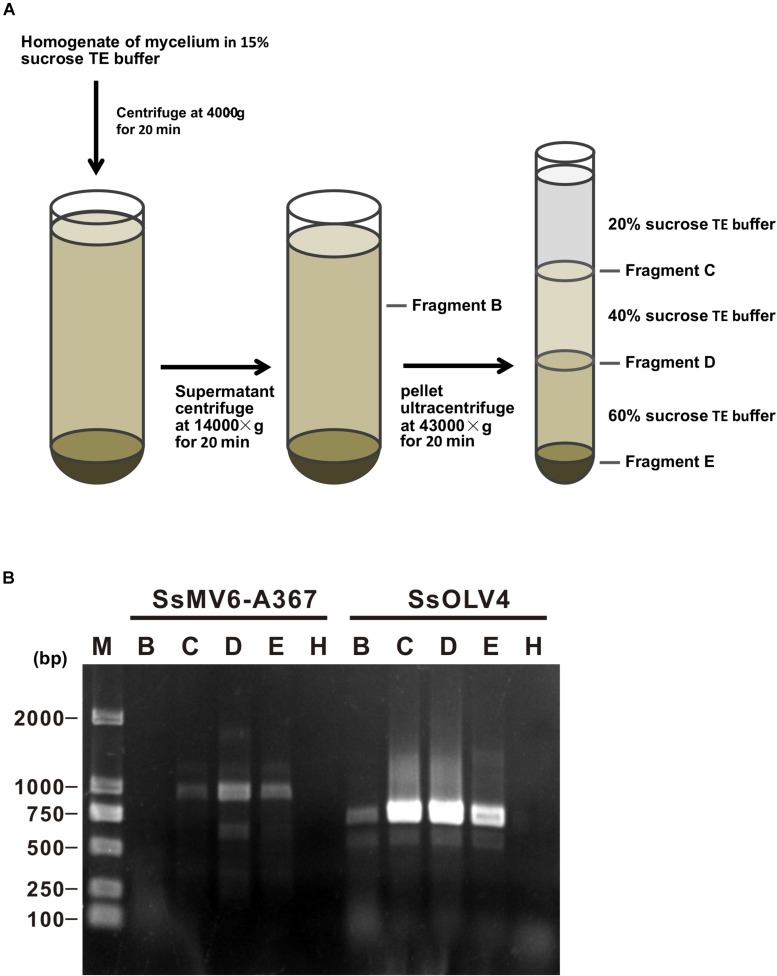
Detection of SsOLV4 and SsMV6-A367 in the cytoplasm and mitochondria of strain Ep-1PNA367P. Each fraction was purified and detected using RT-PCR. **(A)** Schematic diagram for isolating mitochondria. **(B)** RT-PCR detection of SsOLV4 (primers OMVF and OMVR) and SsMV6-A367 (primers MTVB400F and MTVB1346R).

We isolated mitochondria from mycelia of strain Ep-1PNA367P using the density-gradient centrifugation (Figure 7A). After fractionation and purification, SsOLV4 was detected in fractions B (the cytoplasm), D (purified mitochondria), C, and E using RT-PCR (Figure 7B). As a control, the mitovirus *S. sclerotiorum* mitovirus 6-A367 was only detected in fraction D. The results suggest that SsOLV4 exists in both cytoplasm and mitochondria.

### SsOLV4 Has Little Effect on Host Growth and Virulence

**FIGURE 8:**
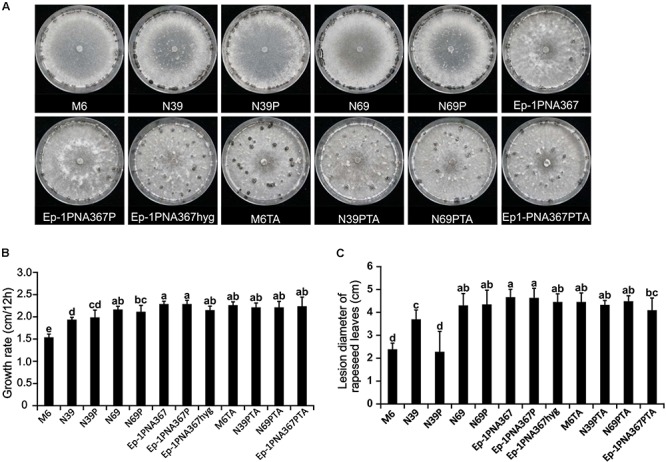
Biological properties of the *S. sclerotiorum* strain artificially infected by SsOLV4. Strains M6, N39, and N69 are the protoplasm-regenerants of strain PX14A4; strains N39P, N69P, and Ep-1PNA367P are the strains transfected by synthesized RNA. A synthesized virus was also transmitted to Ep-1PNA367 through hyphal anastomosis to obtain strains M6TA, N39PTA, N69PTA, and Ep-1PNA367PTA. **(A)** Colony morphology of strains (PDA, 14 days, 21°C). **(B)** Radial mycelial growth rate (PDA, 21°C). **(C)** Lesion diameter on rapeseed leaves (21°C). In each histogram, bars labeled with the same letters are not significantly different (*p* > 0.05) according to the least significant difference test.

The SsOLV4-infected strain PX14A4 and M6 showed stunted growth and weak virulence, and the virus-free strains N39 and N69 somehow recovered with strong virulence and high growth rate. However, when strains N39, N69, and Ep-1PNA367 were infected by SsOLV4, their growth rate and virulence were not significantly influenced, except for strain N39P. The SsOLV4-transmitted strain M6TA, N39PTA, N69PTA, and Ep-1PNA367PTA exhibited similar growth rates and virulence to those of the strain Ep-1PNA367hyg (Figure 8). These findings suggest that SsOLV4 might have little or no impact on the virulence of *S. sclerotiorum*.

## Discussion

### The Genome of Ourmia-Like Mycoviruses Is Different From That of Ourmiaviruses

In this study, we identified a novel ourmia-like mycovirus (SsOLV4) in *S. sclerotiorum*. It is phylogenetically related to viruses in genus *Botoulivirus* in the newly established family *Botourmiaviridae*. According to current taxonomic information from ICTV (2018b.v2), family *Botourmiaviridae* includes four approved genera based on the phylogenetic relationship^2^. Members of the genera (*Botoulivirus*, *Magoulivirus*, and *Scleroulivirus*) are mycoviruses (except for SlAOV1 and SlAOV2, which were isolated from soybean phyllosphere phytobiome, possibly from fungi growing on/in leaves); members in genus *Ourmiavirus* are plant viruses and have segmented genomes (usually harboring three RNA segments) (Crivelli et al., 2011). However, recent reports implied that ourmia-like mycoviruses contain only one RNA segment (Donaire et al., 2016; Hrabakova et al., 2017; Illana et al., 2017; Li et al., 2019). In this study, the synthesized RNA of SsOLV4 could replicate in and transfer among *S. sclerotiorum* strains via hyphal anastomosis, suggesting that only the RdRp-coding RNA segment is sufficient for SsOLV4 replication initiation and transmission. In this regard, ourmia-like viruses are significantly different from ourmiaviruses.

### Features of Termini in Viruses in *Botourmiaviridae* and Other Related Families

Interestingly, we found that SsOLV4 has a G-pentamer at the 5′-terminus and a C-pentamer at the 3′-terminus. Similar tetramers have been found in *Saccharomyces 20S RNA narnavirus* (NC_004051.1) and *Saccharomyces 23S RNA narnavirus* (NC_004050.1) (Figure 2B). The G tract and complementary structure at the termini of RNA could cyclize linear RNA, which may inhibit the digestive activity of exonucleases, thus help viruses escape from the monitoring of antiviral defenses (Tucker and Parker, 2000; Stevens, 2001). Similar terminal structures could be found in the genomes of mitoviruses (G-tetramer or G-trimer at the 5′-terminus and, correspondingly, C-tetramer or C-trimer at the 3′-terminus), and ourmiaviruses (C-trimer at the 5′-terminus, and C-trimer at the 3′-terminus) (Table 1). The diversity of genome termini among viruses in *Botourmiaviridae* or in narna-like viruses, combining the various lengths of 5′- and 3′-UTRs, suggests that viruses have adapted to various hosts. Nonetheless, we have not ruled out that the genome sequences of some viruses are not truly complete or correct (many viral sequences contain complete CDS).

### SsOLV4 Exists in Cytoplasm and Mitochondria

Mitochondria use both UGG and UGA as tryptophan (Try) codons with a few exceptions (Yamao et al., 1985). However, in the *Rhizoctonia* mitovirus 1 and *Rhizoctonia* M2 virus genomes, no tryptophan amino acid was encoded by UGA codons, indicating viral genomes could be translated into a putative polypeptide using either mitochondrial or universal genetic codons, and the viruses are proved to locate in both the cytoplasm and mitochondria (Lakshman et al., 1998; Das et al., 2016). The RNA of mitovirus is not infectious when mixed with host protoplasts (Hillman and Cai, 2013), and mitoviruses are usually difficult to eliminate from their hosts (Wu et al., 2016). Although ourmia-like virus PlRV1 is retained mainly in the mitochondrial fraction, it also retains a small portion in the cytoplasm (Hrabakova et al., 2017). We found that SsOLV4 could be eliminated from strain PX14A4 and that synthesized viral RNA of SsOLV4 could successfully infect the protoplasts. Most importantly, we detected SsOLV4 in both cytoplasm and mitochondria. Since there is not any Try coded by UGA in each unique ORF of ourmia-like viruses (SsOLV4, PlRV1, AnOLV1, and PoOLV1), these viruses may have the ability to express active proteins in both the cytoplasm and mitochondria. The location feature of SsOLV4 was similar with some mitoviruses, but the infectious ability is similar to narnaviruses (Esteban and Fujimura, 2003). This result supports that the ourmia-like mycoviruses are different from viruses in *Narnaviridae*.

### Possible Origin of SsOLV4 and Other Fungal Ourmia-Like Viruses

The putative RdRps of ourmiaviruses share a distant similarity with fungal viruses in family *Narnaviridae*. One hypothesis is that the ourmiaviruses have evolved by reassortment of genomic segments of RNA viruses infecting hosts belonging to different eukaryotic kingdoms, in particular, fungi and plants (Rastgou et al., 2009). However, we cannot ascertain the direction of the viral cross-kingdom transfer. Although the cell wall has been proven to limit the molecules entering fungi (Wang et al., 2016), cytoplasm leakage often occurs in fungi, especially in attenuated strains (Xiao et al., 2014); moreover, fungi often secrete many enzymes to degrade plant cell walls (Inglis and Kawchuk, 2002; Ten Have et al., 2002). Our results prove that the RNA of SsOLV4 could transfect mycelia in some conditions, which may be an example for the hypothesis.

### Replication of SsOLV4

When we compare the viral sequences of SsOLV4 in strains Ep-1PNA367RA, Ep-1PNA367P, and Ep-1PNA367SPE to those in pMD18-P1 (Supplementary Figure S4B), we found only a few mutants (two or three mutants for each strain). The viral termini existed in the sense and negative strands of virus RNA in strain M6 and the transfected strains (Ep-1PNA367P, Ep-1PNA367SPE, and Ep-1PNA367RA). Furthermore, there was no effect on infectivity when excess nucleotide bases were added in the 3′-terminus of the virus. SsOLV4 seems to have a mechanism to correct the redundant sequence of its termini. A similar phenomenon exists in yeast 23S RNA virus (or *Saccharomyces 23S RNA narnavirus*) (Fujimura and Esteban, 2004). This feature suggests that the initiation of SsOLV4 replication is similar to that of narnaviruses in yeast.

## Materials and Methods

### Fungal Strains and Culture Conditions

*Sclerotinia sclerotiorum* strain PX14A1 and PA14A4 was originally isolated from a sclerotium sample on a diseased stem of rapeseed (*Brassica napus*) plants in Sichuan Province, China; strain Ep-1PNA367 is a virulent strain of *S. sclerotiorum* and harbors a virus *S. sclerotiorum* mitovirus 6-A367. Ep-1PNA367hyg is a hygromycin-resistant strain of Ep-1PNA367. All strains and their derivatives were cultured on PDA at 21°C and stored at 4°C.

### Protoplast Preparation and Viral Elimination

The protoplast preparation followed the method described previously. Briefly, 7 g of fresh mycelia was put into a 20-ml digestion buffer with 0.8 M MgSO_4_, 0.1% (w/v) Snailase (Kerui, Wuhan, China) and 1% (w/v) lysing enzymes from *Trichoderma* (Sigma-Aldrich, St. Louis, United States). After 3 h of digestion at 30°C, the protoplasts were collected by centrifugation for 10 min at 4,000 × *g*. The protoplast-containing precipitate was resuspended with 1 ml of STC (1 M sorbitol, 50 mM Tris–HCl, and 50 mM CaCl_2_, pH 8.0) and diluted with STC to 1 × 10^4^ cells per milliliter (for transfection) or 1 × 10^2^ cells per milliliter (for elimination). Protoplasts were regenerated on RM medium [0.7 M sucrose, 1% (w/v) yeast extract] at 21°C.

Strain PX14A4 was subcultured three times by picking hyphal tips on an antiviral PDA medium containing 0.5 mg/ml (w/v) of ribavirin and 0.1 mg/ml (w/v) of cycloheximide. Then the subcultured mycelia were placed onto an antiviral PDA plate covered with cellophane and incubated at 21°C for 48 h. The edge of the colony was harvested and used to prepare the protoplast. After the protoplasts were cultured for 24 h, the regenerated colony was poured onto a fresh PDA.

### Extraction and Purification of dsRNA and Total RNA

The procedure for dsRNA extraction was described previously (Xie et al., 2006). Briefly, strains grew for 3 to 5 days on cellophane membranes placed on top of the PDA medium. Fresh mycelia (200 mg) were harvested and ground to fine powder in liquid nitrogen to isolate dsRNA. The dsRNA was treated with S1 nuclease and DNase I (Takara Bio Inc., Dalian, China) following the manufacturer’s instructions. The final dsRNA fraction was electrophoresed in a 1% agarose gel and visualized by staining with ethidium bromide. The dsRNA segments were isolated and purified from agarose gel using a gel extraction kit (Axygen Scientific Inc., Wujiang, China).

To extract total RNA, strains PX14A1 and PX14A4 were allowed to grow on top of a cellophane membrane overlying a PDA plate for 3 days, and about 100 mg of fresh mycelia were harvested and ground to fine powder in liquid nitrogen with a mortar and pestle. Total RNA was prepared by using a NI-*S. sclerotiorum* RNA reagent (NEWBIO INDUSTRY, Wuhan, China) according to the manufacturer’s instructions.

### NGS and Bioinformatics Analyses

Ribosomal RNA depletion, library preparations, and Illumina sequencing were performed by Personalbio Company (Shanghai, China). The paired-end sequence reads were trimmed by Trimmomatic (0.39) (Bolger et al., 2014) and *de novo* assembly by Trinity (v2.9.0) (Grabherr et al., 2011). Assembled contigs were compared against the NCBI nr database using BLASTX (Altschul et al., 1990). The reads mapping was performed by Bowtie2 (Langmead and Salzberg, 2012). The mapping result visualization was performed by Integrative Genomic viewer (IGV) (Thorvaldsdottir et al., 2013).

### Molecular Cloning and Sequencing

The cDNA synthesis and sequencing of dsRNAs were conducted as previously described (Xie et al., 2006). Based on the sequence of contig DN382_c0_g1_i3, which was similar to the sequence of PlRV1, the specific PCR primer pairs were designed with Primer Premier 5 software (Lalitha, 2000). RT-PCR was performed as previously described (Liu et al., 2009) with slight modifications. Briefly, 1,000 ng total RNA was treated with Recombinant DNase I (RNase-free) (Takara) and heated for 2 min at 80°C to inactivate the DNase I. The reverse transcription was performed using Reverse Transcriptase M-MLV (RNase H-) (Takara) and ribonuclease inhibitor (Takara). PCR amplification was performed using 2 × EasyTaq PCR SuperMix (+dye) (Transgen, Beijing, China) or FastPfu DNA polymerase (Transgen), including an initial denaturation step of 3 min at 95°C, followed by 31 cycles of 30 s at 94°C, 30 s at 58°C, 1–3 min at 72°C, with a final elongation step of 5 min at 72°C. PCR products were fractionated by gel electrophoresis and stained with ethidium bromide for imaging. The target segment was separated and purified with an extraction kit (Axygen Scientific Inc.). Each purified segment was ligated to the pMD18-T vector (Takara) and sequenced three times.

To determine the terminal sequences of SsOLV4, single-primer-dependent amplification following a method previously described was used (Potgieter et al., 2009), and classical RACE (Scotto-Lavino et al., 2006) was used to confirm the terminal sequence. The obtained sequences were assembled using DNAMAN (version 8) software with a minimum overlap length of 40 bp with an identity of over 90%.

### Construction of Full-Length Viral Clones and Different Templates

The full-length cDNA clone of the SsOLV4 genome was constructed from two overlapping cDNA fragments by RT-PCR from total RNA extracted from strain M6 (shown in Supplementary Figure S1 and primers listed in Supplementary Table S3). The DNA segment T7A was amplified using primer T7-OMFA (with the T7 core promoter sequence in the 5′-terminus) and OMR1. The DNA segment, named HMA, was amplified using the primer OMF2 and OMRA-Ham (with the hammerhead ribozyme sequence at the 3′-terminus). These two segments were then purified and ligated into vector pMD18-T, respectively. The plasmid was transformed into *Escherichia coli* strain TOP10 by heat shock. The clones were confirmed by Sanger sequencing. The plasmids pMD18-T7A2 and pMD18-HMA2 had the correct direction and almost the right sequences. A 1.9-kb segment was obtained from the *Sal*I and *Apa*I digested plasmid pMD18-T7A. A 3.7-kb segment was obtained from *Sal*I and *Apa*I digested plasmid pMD18-HMA. The two pieces of segment were ligated by T4 DNA ligase (Takara) to obtain the plasmid pMD18-P1, which contains full-length SsOLV4 cDNA with a T7 promoter in the 5′-terminus and a hammerhead ribozyme in the 3′-terminus (Supplementary Figure S2B).

### *In vitro* Transcription and Transcript Transfection

To obtain complete infectious RNA, T7 polymerase was used for the transcription test *in vitro*. The method followed the protocol of RiboMAX Large-Scale RNA Production Systems-T7 (Promega Corporation, WI, United States). Three kinds of DNA were used as templates (Supplementary Figure S2B), namely, PCR products of the primer pair M13 (+) and OMRA based on the plasmid pMD18-P1 or linearized plasmid P1 (digested by *Bam*HI or *Spe*I). The synthesized viral RNA was used to transfect the *S. sclerotiorum* protoplasts as previously described (Marzano et al., 2015). We used 1.14-μg template DNA (0.1 μg/μl of purified DNA, 11.4 μl) to synthesize RNA (5.6 μg/μl RNA, 25 μl obtained). Ten microliters of synthetic RNA was added to 50 μl of protoplasts and mixed thoroughly to get mixture A. Mixture A was kept on ice for 30 min, and then 400 μ μl of PEG buffer [60% (w/v) PEG4000, 50 mM Tris–HCl, 50 mM CaCl_2_, pH 7.5] was added to get mixture B. Mixture B was poured onto the RM medium and incubated at 21°C for 4 days. The colony was poured out to a fresh PDA medium (Supplementary Figure S3B) and subcultured three times. For transfection of the mycelia, the synthetic RNA was mixed with PEG buffer and added dropwise onto the edge of the fungal colonies grown on PDA plates. The mycelia outside were transferred to new PDA plates for subculture (Supplementary Figure S3A).

### Northern Blot and Dot Blot Hybridization

Fresh mycelia cultured for 3 days were used to extract the total RNA. The probe is a 747-bp segment amplified with pMD18-P1 as a template and the OMFA/OMR2 as primers. The northern blot was carried out following the manual of alkaline Direct Labeling System and Amersham Hybond-N+ (GE Healthcare, Little Chalfont, United Kingdom). For northern blot, total RNA was denatured for 10 min at 70°C and separated using denatured agarose gel with formaldehyde and then transferred to a positively charged nylon membrane (GE) using alkaline transfer solution (0.01 M NaOH, 3 M NaCl). For the dot blot, RNA samples (2 μ μl) were added on the positively charged nylon membrane and dried out. The dried nylon membrane was cross-linked using a UV cross-linker. The hybridization was performed overnight in a glass tube at 55°C. The chemiluminescent signal generation and detection were performed using an Amersham CDP-Star Detection Reagent (GE).

### Sequence Similarity Search and Phylogenetic Analyses

A sequence similarity search was performed using BLASTX (Altschul et al., 1997) against the NCBI RefSeq database. Multiple alignment and phylogenetic analyses of protein sequences were constructed using MEGA7 (Kumar et al., 2016).

### Detection of SsOLV4 in the Mitochondria and Cytoplasm

We isolated cytoplasm and mitochondria (Figure 7A) following the method described previously (Wu et al., 2016) with slight modifications. Briefly, mycelia of strain Ep-1PNA367P (about 10 g) were harvested and mixed with 50 ml of 15% sucrose (w/v) in Tris–EDTA (TE) buffer (100 mM Tris–HCl and 0.2 mM EDTA, pH 7.4) and homogenized with a Dounce tissue homogenizer. The mixture was filtrated with gauze to remove the big residue, and the filter liquor was centrifuged at 4,000 *g* for 20 min at 4°C to remove mycelial fragment and nuclei. The supernatant was centrifuged at 14,000 × *g* for 20 min at 4°C. The pellet was resuspended with 2 ml of 20% sucrose (w/v) in TE buffer and centrifuged with sucrose gradient. Each fraction was collected separately and used for extraction of total RNAs. RT-PCR was used to detect the viruses in different fractions.

### Mycelial Growth and Virulence Test

To evaluate the effect of SsOLV4 on colony morphology and the virulence of *S. sclerotiorum* to rapeseed, SsOLV4-infected and SsOLV4-free strains (Ep-1PNA367 and Ep-1PNA367hyg) and regenerant strains (N39 and N69) were inoculated on leaves of rapeseed following the methods previously described (Zhang et al., 2009). Each test had five repeats, and data from the experiments were analyzed using an analysis of variance in the SPSS software (IBM SPSS Statistics 19). Treatment means were compared with the test of Duncan at the *p* = 0.05.

## Data Availability Statement

This article contains previously unpublished data. Sequence file of SsOLV4 is available from the NCBI, GenBank Accession No. MN715322.

## Author Contributions

DJ and QW designed the research. QW, YF, and DJ wrote the manuscript. QW and FM executed the experiments. QW, FM, JC, and JX performed the data and bioinformatics analyses. All authors read and approved the final manuscript.

## Conflict of Interest

The authors declare that the research was conducted in the absence of any commercial or financial relationships that could be construed as a potential conflict of interest. The handling Editor declared a past co-authorship with the authors JX and DJ.

## Footnotes

1 http://nls-mapper.iab.keio.ac.jp/

2 https://talk.ictvonline.org/taxonomy/
