# Supplementary material for: A Single ssRNA Segment Encoding RdRp Is Sufficient for Replication, Infection, and Transmission of Ourmia-Like Virus in Fungi
Source: Front Microbiol. 2020 Mar 18;11:379. doi: 10.3389/fmicb.2020.00379 (PMC7093599; doi:10.3389/fmicb.2020.00379)
Supplement: Supplementary file 1 [file Data_Sheet_1.pdf]

Supplementary Material

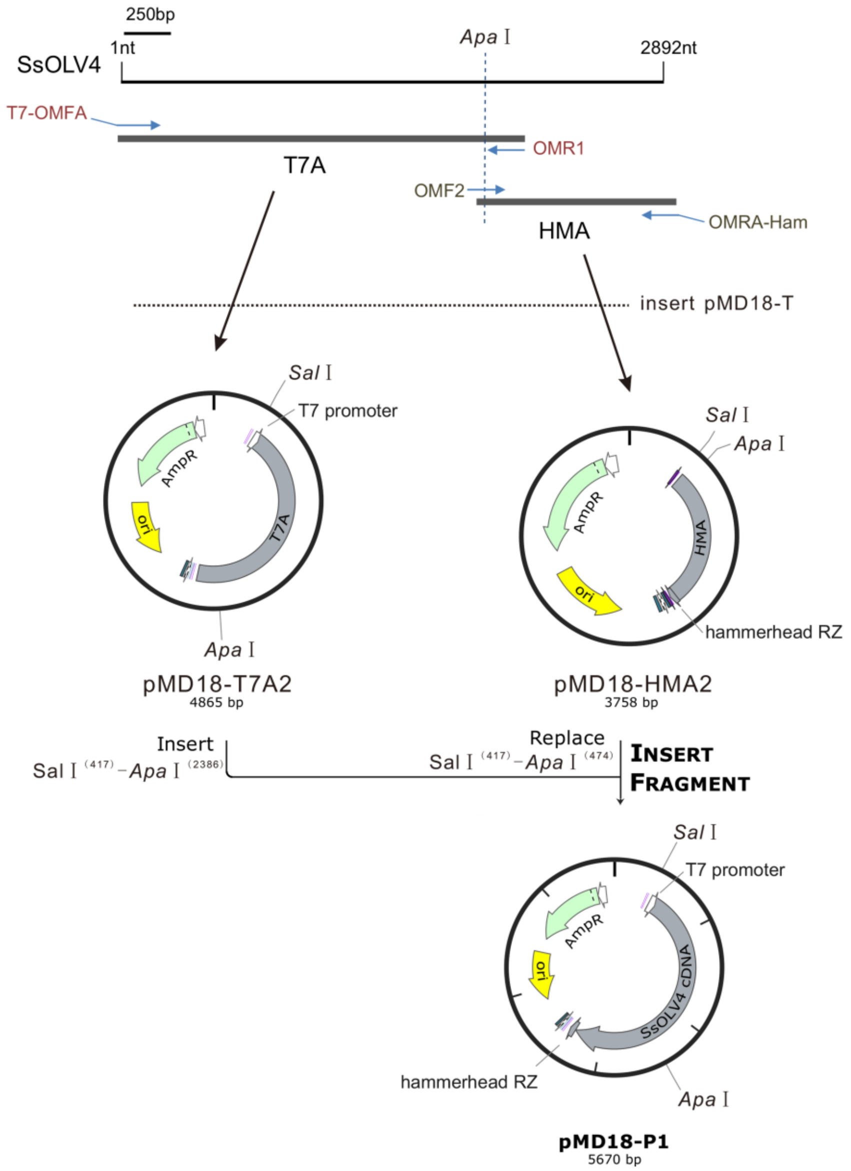

FIGURE S1 The schematic diagram for constructing the full-length infectious cDNA clone of SsOLV4. The full-length cDNA clone of the SsOLV4 was constructed from two overlapping cDNA fragments by RT-PCR from total RNA extracted from strain M6. Two DNA segments T7A (amplified using primers T7-OMFA and OMR1) and HMA (amplified using primers OMF2 and OMRA-Ham) were ligated into vector pMD18-T (Takara) to obtain plasmids pMD18-T7A2 and pMD18-HMA2. These two plasmids were treated with *Sal* I and *Apa* I to obtain a 1.9 kb segment and a 3.7 kb segment. These two segments were ligated by T4 DNA ligase (Takara) to obtain the plasmid pMD18-P1.

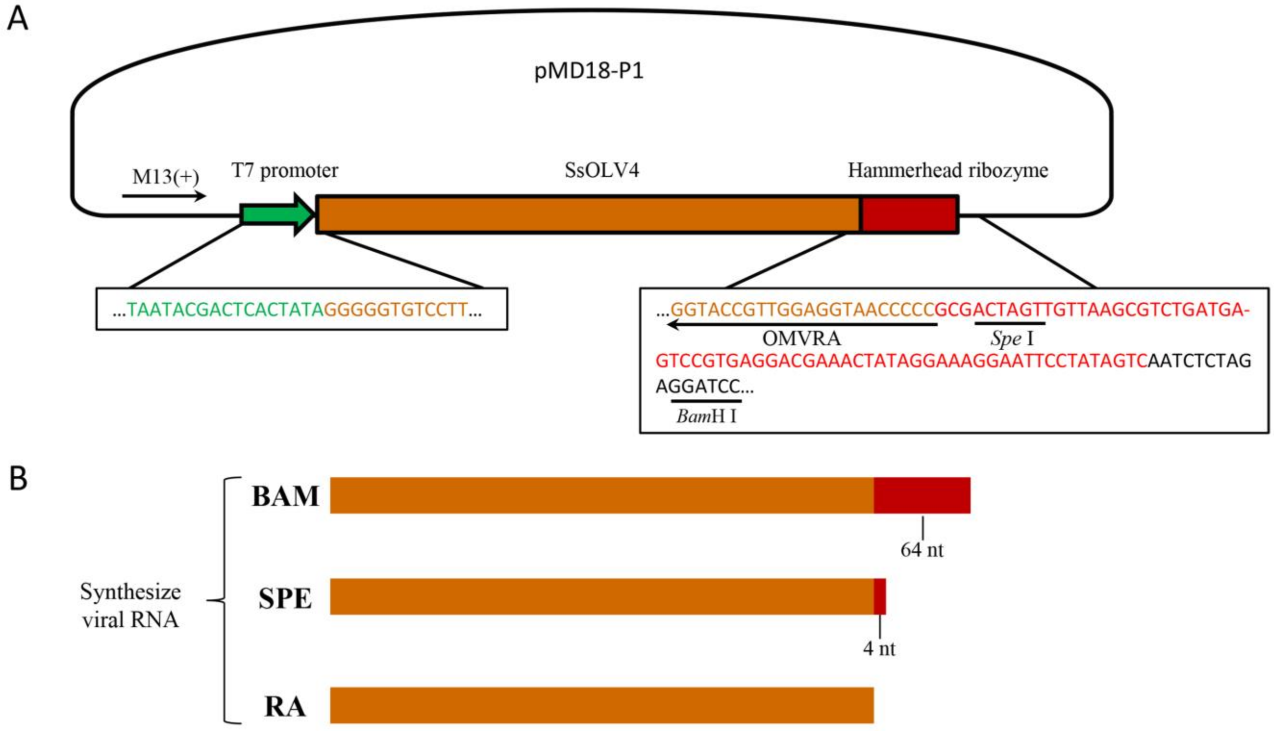

FIGURE S2 The schematic diagram for different template used for transcrption *in vitro*. (A) Three DNA fragments were used as templates. The *Bam*H I or *Spe* I linearized plasmid pMD18-P1 and a DNA fragment amplified using primers M13(+) and OMVRA (sequence listed in Table S3). The green arrow and sequence indicate T7 promoter. The orange boxes indicate sequence of full-lentgh cDNA of SsOLV4. The red boxed indicate the hammerhead ribozyme. The black line indicates sequence belonging to vector pMD18-T. The enzyme digest regions (*Bam*H I and *Spe* I) were underlined. The primers M13(+) and OMVRA was indicated with black arrow. (B) T7 RNA polymerase was used for the transcription *in-vitro*. RNA "BAM" and RNA "SPE" was synthesized based on templates *Bam*H I or *Spe* I linearized plasmid pMD18-P1. RNA "RA" was synthesized based on the DNA fragment amplified using primers M13(+) and OMVRA. The excess sequence numbers were indicated.

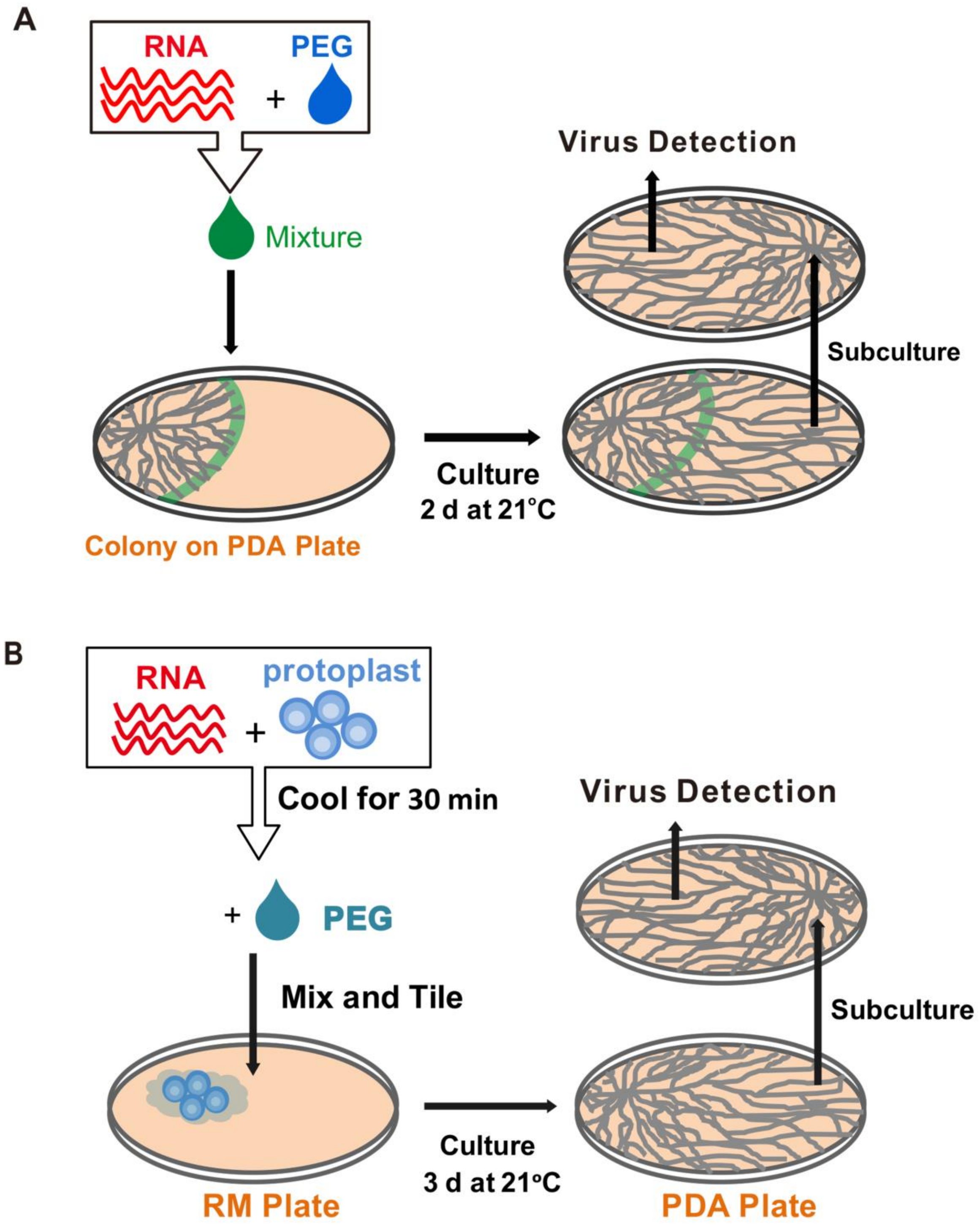

FIGURE S3 The schematic diagram for synthesize viral RNA transfection of mycelia or protoplasts. (A) 10 uL of synthesize viral RNA was mixed with 10 uL PEG buffer and added to the tips of mycelia and the colony was cultured for 2 d at 21°C. (B) 10 uL of synthesize viral RNA was mixed with 50 uL of protoplasts thoroughly and cooled on ice for 30 min. Then 400 uL PEG buffer was added to the cold mixture. Final the mixture was added on RM medium plate and cultureed for 4 d at 21°C. The newly formed mycelia was picked to the fresh PDA plate and subcultured for 3 times or more.

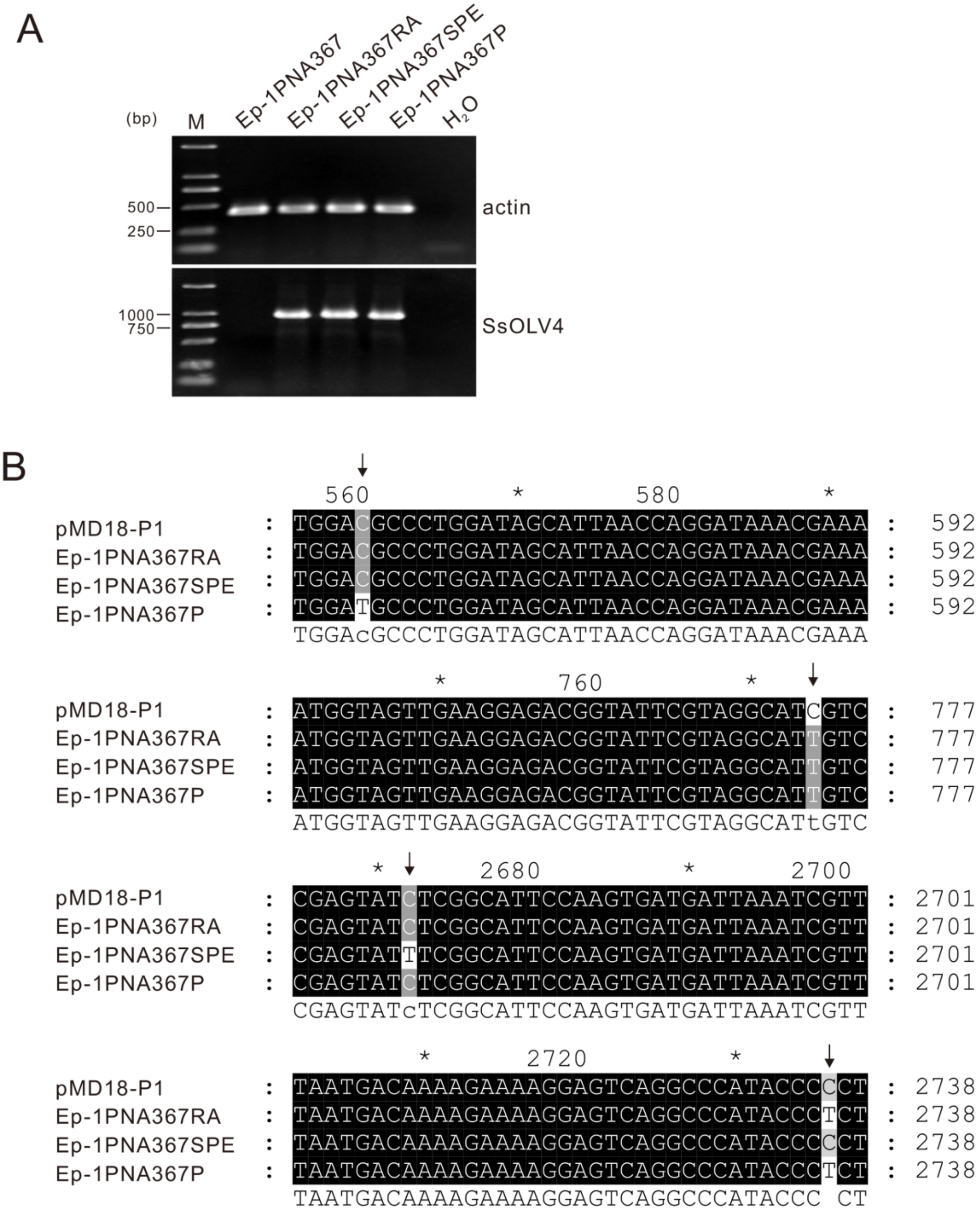

FIGURE S4 Comparison of the SsOLV4 sequence in transfected strains of *S. sclerotiorum*. (A) RT-PCR amplification was used to examine SsOLV4 in strains Ep-1PNA367RA, Ep-1PNA367SPE and Ep-1PNA367P, which were the protoplast-regenerants of Ep-1PNA367 transfected by viral RNA synthesized based on the PCR production or linearized plasmid pMD18-P1 digested by *Bam*H I or *Spe* I. *Actin* was amplified as an internal control. The primers are listed in Table S1. The marker M was DL2000 (Takara). Size of DNA ladder standard is indicated in bp. (B) Comparison of the sequences of SsOLV4 in regenerants to the viral full-length cDNA in pMD18-P1. The number illustrates the positions of the viral sequence.
