# Supplementary material for: A Single ssRNA Segment Encoding RdRp Is Sufficient for Replication, Infection, and Transmission of Ourmia-Like Virus in Fungi
Source: Front Microbiol. 2020 Mar 18;11:379. doi: 10.3389/fmicb.2020.00379 (PMC7093599; doi:10.3389/fmicb.2020.00379)
Supplement: Supplementary file 2 [file Table_1.docx]

**Table S1 Primer pairs used to identity the virus in each strains**

| **Primer** | **Primer sequence (5’ →3’)** | **Application** | **Product size (bp)** |
| --- | --- | --- | --- |
| MTVB400F | GGTCGTTTCAGCAAAGTCGGTC | Detect Sclerotinia sclerotiorum mitovirus 6-A367 (SsMV6-A367) | 947 |
| MTVB1346R | GGAAGACGATCGGTAGCAGCAC |  |  |
| ActinF | CTGGAAGATTGACTGGCGGTTTG | Used as an internal control | 419 |
| ActinR | AGCACCAGAGGAGCACCAGTTT |  |  |
| HygF | TCACTGGCAAACTGTGATGGA | Detect hygromycin resistance gene | 218 |
| HygR | AGATGTTGGCGACCTCGTATT |  |  |
