# Supplementary material for: A Single ssRNA Segment Encoding RdRp Is Sufficient for Replication, Infection, and Transmission of Ourmia-Like Virus in Fungi
Source: Front Microbiol. 2020 Mar 18;11:379. doi: 10.3389/fmicb.2020.00379 (PMC7093599; doi:10.3389/fmicb.2020.00379)
Supplement: Supplementary file 3 [file Table_2.docx]

**Table S2 Predicted NLSs in SsOLV4 RdRp**

| **Position** | **Sequence** | **Type** |
| --- | --- | --- |
| 73 | RARETKRRRSAEE | Monopartite NLS |
| 121 | RLRYHKRNHLISNVGF | Monopartite NLS |
| 699 | QLLKRRRAV | Monopartite NLS |
| 73 | RARETKRRRSAEEEREGAAKSGRE | Bipartite NLS |
