# Supplementary material for: A Single ssRNA Segment Encoding RdRp Is Sufficient for Replication, Infection, and Transmission of Ourmia-Like Virus in Fungi
Source: Front Microbiol. 2020 Mar 18;11:379. doi: 10.3389/fmicb.2020.00379 (PMC7093599; doi:10.3389/fmicb.2020.00379)
Supplement: Supplementary file 4 [file Table_3.docx]

**Table S3 Primers used for SsOLV4 cDNA amplification and sequencing**

| **Primer** | **Primer sequence (5’→3’)** | **Position** |
| --- | --- | --- |
| OMFA | GGGGGTGTCCTTACGGACTCTG | 1-22 |
| T7-OMFA | **TAATACGACTCACTATA**GGGGGTGTCCTTACGGACTCTG | 1-22 |
| OMF | AAGGTGCCGCCAAGTCA | 676-692 |
| OMF1 | GAACTGGCTGACTTTGGTATCG | 1227-1248 |
| OMF2 | GGTACCAGTAGAGGTGACCTTGT | 1896-1918 |
| OMF3 | TATCGGAAATGTACGGTCTGTCG | 2644-2666 |
| OMR2 | ACGAATACCGTCTCCTTCAACTAC | 767-744 |
| OMR | GGCTTACCCGAGGAATGAAC | 1411-1392 |
| OMR1 | AAGCGTTATTCGTTACTACCATCC | 2154-2131 |
| OMRA | GGGGGTTACCTCCAACGGTACC | 2892-2871 |
| OMRA-Ham | GACTATAGGAATTCCTTTCCTATAGTTTCGTCCTCACGGACTCATCAGACGCTTAACAACTAGTCGC  GGGGGTTACCTCCAACGGTAC | 2892-2871 |
| M13(+) | GGTTTTCCCAGTCACGACGTTG |  |

Nucleotides bolded represent the T7 promoter sequence.

Nucleotides underlined in the OMRA-Ham primer represent hammerhead ribozyme sequence.
